# Respiratory symptoms and lung function in patients treated for pulmonary tuberculosis in Malawi: a prospective cohort study

**DOI:** 10.1136/thoraxjnl-2021-217190

**Published:** 2021-12-22

**Authors:** Rebecca Nightingale, Beatrice Chinoko, Maia Lesosky, Sarah J Rylance, Bright Mnesa, Ndaziona Peter Kwanjo Banda, Elizabeth Joekes, Stephen Bertel Squire, Kevin Mortimer, Jamilah Meghji, Jamie Rylance

**Affiliations:** 1 Department of Clinical Sciences, Liverpool School of Tropical Medicine, Liverpool, UK; 2 Liverpool Univeristy Hospitals NHS Foundation Trust, Liverpool, UK; 3 Malawi Liverpool Wellcome Trust Clinical Research Programme, Blantyre, Malawi; 4 Division of Epidemiology & Biostatistics, University of Cape Town, Rondebosch, South Africa; 5 Department of Medicine, Queen Elizabeth Central Hospital, Blantyre, Malawi

**Keywords:** tuberculosis

## Abstract

**Rationale:**

Pulmonary tuberculosis (PTB) can cause post-TB lung disease (PTLD) associated with respiratory symptoms, spirometric and radiological abnormalities. Understanding of the predictors and natural history of PTLD is limited.

**Objectives:**

To describe the symptoms and lung function of Malawian adults up to 3 years following PTB-treatment completion, and to determine the evolution of PTLD over this period.

**Methods:**

Adults successfully completing PTB treatment in Blantyre, Malawi were followed up for 3 years and assessed using questionnaires, post-bronchodilator spirometry, 6 min walk tests, chest X-ray and high-resolution CT. Predictors of lung function at 3 years were identified by mixed effects regression modelling.

**Measurement and main results:**

We recruited 405 participants of whom 301 completed 3 years follow-up (mean (SD) age 35 years (10.2); 66.6% males; 60.4% HIV-positive). At 3 years, 59/301 (19.6%) reported respiratory symptoms and 76/272 (27.9%) had abnormal spirometry. The proportions with low FVC fell from 57/285 (20.0%) at TB treatment completion to 33/272 (12.1%), while obstruction increased from and 41/285 (14.4%) to 43/272 (15.8%) at 3 years. Absolute FEV_1_ and FVC increased by mean 0.03 L and 0.1 L over this period, but FEV_1_ decline of more than 0.1 L was seen in 73/246 (29.7%). Higher spirometry values at 3 years were associated with higher body mass index and HIV coinfection at TB-treatment completion.

**Conclusion:**

Spirometric measures improved over the 3 years following treatment, mostly in the first year. However, a third of PTB survivors experienced ongoing respiratory symptoms and abnormal spirometry (with accelerated FEV_1_ decline). Effective interventions are needed to improve the care of this group of patients.

**Key messages**

**What is the key question?**

- Following successful treatment for pulmonary tuberculosis (TB), ongoing respiratory symptoms and abnormal lung function are well recognised: how do these change over time?

**What is the bottom line?**

- We found that among adults in Malawi who had completed treatment for pulmonary TB, the majority experienced improvement including an increase in absolute FVC by over 0.1 L, mostly in the first year. However, at 3 years after treatment completion, one in five still had significant respiratory symptoms and more than one quarter had abnormal spirometry.

**Why read on?**

- Discover the rates of change in spirometry, the possible determinants and recommendations for the future.

## Background

In 2019, there were an estimated 10 million new cases of tuberculosis (TB) worldwide, of which a quarter occurred in sub-Saharan Africa.^1^ The WHO estimates the incident rate of TB in Malawi is 146 per 100 000 population, with 27 000 new cases in 2019.^2^ Over the last decade, TB survival has improved, in part due to higher treatment completion rates (currently 85%), and demonstrates how effective treatment can be.^1^ However, as a result, more people are living with long-term health consequences of having had TB disease and receiving TB treatment.^3 4^ The importance of health and well-being post-TB was highlighted during a recent international symposium which identified major gaps in our understanding and proposed a definition of post-TB lung disease (PTLD) as ‘evidence of chronic respiratory abnormality, with or without symptoms, attributable at least in part to previous pulmonary tuberculosis (PTB)’.^3^

We have previously published a study describing the burden and of PTLD in adults after successfully completing PTB treatment in urban Malawi.^5^ This study showed that among 405 individuals (mean age 35 years; 77% microbiologically confirmed PTB; 61% HIV-positive) at TB-treatment completion, 61%, 34% and 44% had respiratory symptoms, abnormal spirometry and bronchiectasis, respectively. In the year after treatment completion, the proportion of participants with respiratory symptoms and abnormal spirometry decreased, but a substantial minority of participants in fact experienced deteriorating lung function over time (FVC fell in 14% and FEV_1_ fell in 19.3%).^5^ These findings were limited by the short follow-up period of 1 year only. To the best of our knowledge, no literature describes the natural history of PTLD for longer than 12 months after treatment completion, but knowing the trajectory of lung disease in this population may help guide clinicians and policy makers alike. In this paper, we describe findings of the extended follow-up this cohort for a further 2 years, to give a detailed description of the natural history of PTLD as defined by respiratory symptoms, spirometry and other health and well-being outcomes, in the period after TB treatment completion.

## Methods

### Study design

We conducted a prospective longitudinal cohort study of adult PTB survivors, enrolled at completion of PTB treatment and followed up for 3 years. Details of the initial cohort design have been published elsewhere.^5^ Screening and recruitment were completed between February 2016 and April 2017, with the final 3-year follow-up being completed in March 2020.

### Setting

Participants were recruited from nine health centres in Blantyre, the second largest city in Malawi, with study visits conducted at Queen Elizabeth Central Hospital. Domiciliary visits were conducted throughout the study if required for logistical reasons.

### Participants

Sequential adults were prospectively identified at pulmonary TB treatment completion and were eligible for inclusion if they were 15 years or older, lived in Blantyre, and had been treated for a first episode of drug susceptible PTB with cure or completion as defined by the National Treatment Programme.^5^ Participants completing year one of study follow-up were invited to continue into a longer-term study with a further 2 years of follow-up. Participants gave written informed consent.

### Procedures

Baseline data from TB-treatment completion and 1-year follow-up are available from the previously reported study.^5^ These include HIV-status and CD4 counts, plain chest X-rays (CXR) taken at TB-treatment completion and 1 year, and non-contrast high-resolution computer tomography (HRCT) scans at TB-treatment completion.

Study visits were completed approximately 6-monthly for the 3-year period. For all visits, participants completed questionnaires administered by study staff, including demographics, socioeconomic data, respiratory exposures and the St George’s Respiratory Questionnaire (SGRQ). Health seeking and medical history were self-reported but confirmed using the participants’ health passport (a patient-held medical record). Six-minute walk tests and postbronchodilator ATS-standard spirometry (Easy One, NDD) were completed according to international standards^6 7^ by experienced staff with formal spirometry qualifications.

FEV_1_ (forced expiratory volume) and FVC (forced vital capacity) were recorded as absolute volumes, and z-scores calculated using the Global Lung Initiative 2012 (GLI-2012) African reference ranges. Obstruction was defined as FEV_1_/FVC below lower limits of normal (LLN) and Low FVC defined as FVC <LLN with FEV_1_/FVC ≥LLN.^8 9^ Obstructive severity was coded according to the Global Initiative for Chronic Obstruction Lung Disease (GOLD) guidelines^9 10^ (online supplemental table 1E).

10.1136/thoraxjnl-2021-217190.supp1 Supplementary data

Radiology was independently assessed by two radiologists according to a piloted scoring tool based on Fleischner society guidelines^11^ adapted for TB (for derivation, see appendix 3 of Ref. 5).

### Study size

The initial study sample size was based on a precision estimate for PTLD at TB-treatment completion, with a sample of 400 required to estimate PTLD prevalence with ±5% precision with 95% confidence, assuming a true population prevalence of between 10% and 50%. All participants in the initial study were invited to continue the extended follow-up.

### Statistical methods

Continuous variables are reported as mean (SD) or medians (IQR) and categorical data as frequency (%). We assessed for selection bias by comparison of those completing the study with those who were lost to follow-up using χ^2^ and Student t-tests. Individuals completing follow-up were classified into three groups: ‘no change’, ‘improvement’ or ‘decline’ in clinical markers using predefined classifications of change over time.^5^ To account for individual variation in follow-up periods, continuous variables were normalised to the 3-year time point using the exact individual measurement interval (except in the longitudinal model of lung function). We tested the differences in the burden of disease using symptom scores, spirometry and 6 min walk test at TB-treatment completion and 3-year follow-up using McNemar’s and Wilcoxon Sign Rank test.

Associations between prespecified predictors and FEV_1_ and FVC over time were estimated using linear mixed-effects models, fitting a random effect for participant, and adjusting for time from TB-treatment completion as a fixed effect. Time from TB treatment completion was also modelled as piecewise linear and using a natural spline in adjusted mixed effects models to test the hypothesis that improvement occurred mostly within the first year of follow-up (online supplemental tables 8E and 9E). Independent variables were predetermined based on past literature and clinical assumptions (online supplemental figure 1E). Radiological variables were selected based on the expert opinion of two pulmonologists, ensuring that the variables selected would highlight key pathologies. Given variable access to radiology in many low-income and middle-income countries (LMICs), we produced three predictive models for each dependant variable (FEV_1_ and FVC): (1) without radiology; (2) including CXR; (3) including HRCT but not CXR to minimise co-correlation effects.

Data were analysed using Stata V.14.2 statistical software and R V.3.4. Statistical significance was tested at the 5% level.

## Results

**Figure 1:**
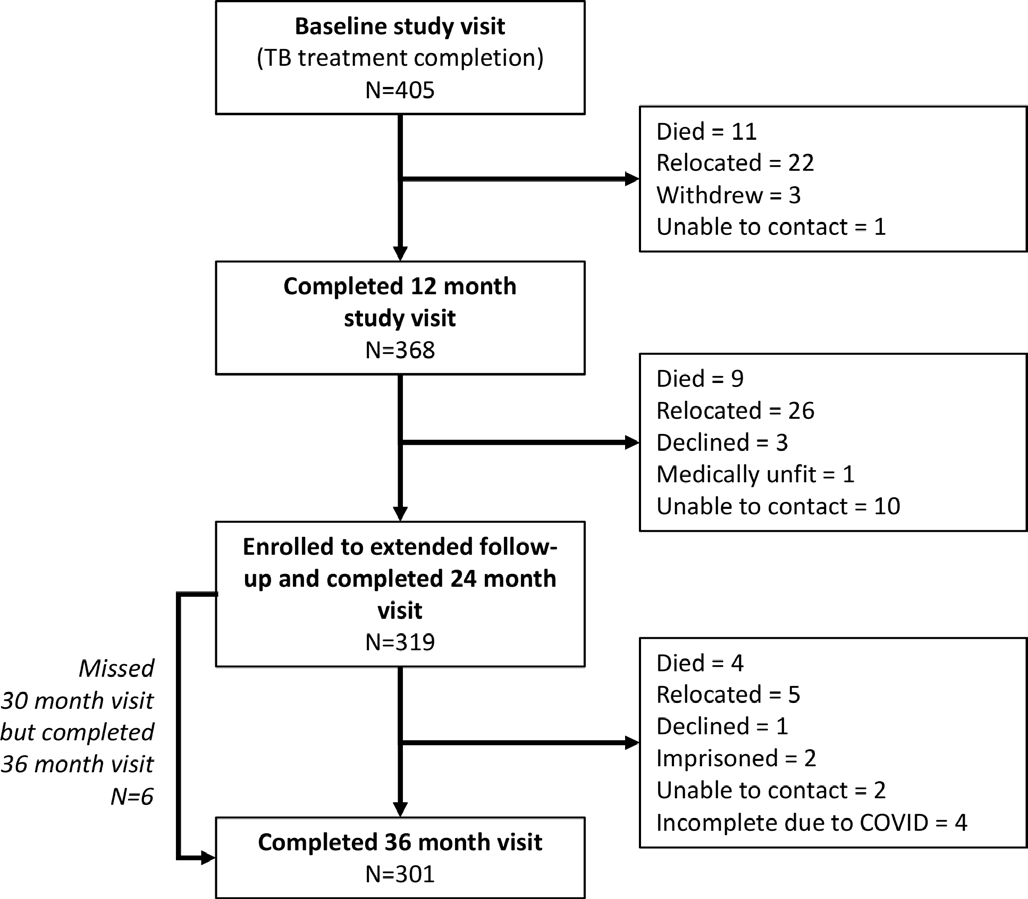
Participant flow diagram.

405 participants were enrolled in the study at TB-treatment completion of whom 368 completed 1-year follow-up,^4^ and 319 participants were enrolled in the extended follow-up (figure 1). Of the 49 participants who did not re-enrol, 37 (75.5%) were male and the mean age was 33.4 years (SD: 9.05). Reasons for not re-enrolling were: death (n=9); relocation (n=26); declined (n=3); medically unfit (n=1); unable to contact (n=10). At 3 years, follow-up data were available for 301 participants. The 18 participants who did not complete the study were due to death (n=4), relocation (n=7), withdrawal (n=1), loss to follow-up (n=2) and premature study closure due to COVID-19 (n=4). Among these participants, sex (n=12/18 male, 67%) and age (mean 34.5 years, SD 7.0) were not significantly different to the overall cohort (p=0.98 and p=0.88, respectively). The median time from TB-treatment completion to the final visit was 1095 (1080–1130) days.

### Participant characteristics

Of participants enrolled to extended 3-year follow-up (n=319), the median age at TB-treatment completion was 35.2 years (SD 10.2 years), and 212 (66.5%) were male (table 1). Among the 192 (60.4%) participants living with HIV, 179 (93.2%) were taking regular antiretroviral medication.

**Table 1:** Participant characteristics at TB treatment completion for the 319 participants who enrolled to the extend follow-up (years 2 and 3 of the study)

Characteristics n=319	Value
Age in years, mean (SD)	35.2 (10.2)
Male sex, n (%)	212 (66.5%)
Microbiological confirmed TB, n (%)	249 (78.1%)
HIV-positive, n (%)	192 (60.4%)
Taking ART (if HIV positive), n (%)	179 (93.2%)
CD4 count, median cells/μL (IQR)	252 (154–407)
Self-reported respiratory condition prior to TB diagnosis, n (%)	138 (43.3%)
Urban SES quintile (n=313), n (%)	
Poorest	20 (6.4%)
2nd poorest	77 (24.6%)
Middle	79 (25.2%)
2nd most wealthy	93 (29.7%)
Most wealthy	44 (14.1%)
Piped water into the dwelling, n (%)	28 (8.8 %)
Share a toilet with another household, n (%)	183 (57.3%)
Has electricity in the household, n (%)	170 (53.3%)
Maximum education ≤primary school, n (%)	128 (40.1%)
Monthly household income, mean US$ (SD)†	40 (60)
Dependent child dropped out of school due to parent’s TB, n (%)	56 (17.6%)
Household dissaving due to TB‡, n (%)	236 (74.0%)
Ever smoked tobacco, n (%)	92 (28.8%)
Tobacco smoking history, median pack-years (IQR)	3 (1–6)
Ever smoked cannabis§, n (%)	46 (15.9%)
Charcoal/wood (biomass) as main fuel, n (%)	306 (95.9%)

Baseline characteristics at initial follow-up have been previously.^4^

*The questioned asked was ‘Before you became unwell with TB, did a doctor/healthcare provider ever tell you that you had a problem with your lungs or breathing?’.

†Missing data: HIV-status n=1—declined test at baseline; SES n=313—unable to confirm housing type in six participants. House income n=225 due to participants not knowing their household income.

‡Borrowed money, took a loan or sold assets in the year prior to TB treatment completion.

§n=289, missing data for 30 participants.

ART, antiretroviral therapy; SES, socioeconomic status; TB, tuberculosis.

Socioeconomic deprivation was high; 191 people (91.2%) did not have piped water to their house, 128 (40.1%) were educated to primary school only or had no education and 236 (74.0%) had experienced dissaving since their TB diagnosis. Tobacco consumption was low: 92 (28.8%) reported having ever smoked, with a median of 3 pack-years consumption (IQR 1–6). Almost all participants used biomass as their main fuel (306, 95.9%).

### Symptoms and other clinical features

The prevalence of respiratory symptoms significantly reduced from treatment completion when 185/319 (60.0%) reported one or more respiratory symptoms (breathlessness, cough, regular sputum or wheeze in the preceding 3 months) compared with 59/301 (19.6%) of participants at 3 years, p<0.001 (table 2). Using the SGRQ tool, the total score fell from median 9 (IQR 1–23) to 0 (IQR 0–1.44), over the same interval, p<0.001. Cough was the most frequently reported residual symptom at the 3-year visit, n=44/301 (14.6%). Most of the reduction in symptom frequency can be attributed to improvement in the first year, with less change in subsequent periods.

**Table 2:** Clinical parameters measured for the 3-year post-TB treatment completion

Variable	TB treatment completion	1-year post-TB treatment completion	2-year post-TB treatment completion	3-year post-TB treatment completion	P value comparing 1-year and 3-year visits*	P value comparing TB completion and 3-year visit*
*Clinical observations*	n=319	n=319	n=319	n=301		
Oxygen saturations, median % (IQR)	98% (97%–99%)	98% (97%–98%)	98% (98%–99%)	99% (98%–100%)	<0.0001	<0.0001
SpO_2_ <92%, n (%)	4 (1.25%)	2 (0.63%)	0 (0%)	0 (0%)	0.1573	0.05
Respiratory rate/minute, median (IQR)	19 (17–20)	20 (19–22)	20 (19–21)	20 (20–21)	0.0193	<0.0001
BMI, median kg/m^2^ (IQR)	20.5 (19–22.3)	21.2 (19.6–23.4)	21.3 (19.6–23.4)	21.3 (19.7–23.9)	0.0168	<0.0001
Self- reported symptoms†					<0.0001	<0.0001
Breathlessness, n (%)	184 (57.7%)	246 (77.12%)	279 (87.5%)	281 (93.4%)		
Never/only with chest infections						
Few days per months	122 (38.2%)	67 (21.0%)	37 (11.6%)	20 (6.6%)		
≥Several days per week	13 (4.1%)	6 (1.88%)	3 (0.9%)	0 (0.0%)		
Cough, n (%)					0.2763	<0.0001
Never/only with chest infections	207 (64.9%)	265 (83.1%)	250 (78.4%)	257 (85.4%)		
Few days per months	102 (32.0%)	48 (15.05%)	56 (17.6%)	41 (13.6%)		
≥Several days per week	10 (3.13%)	6 (1.88%)	13 (4.1%)	3 (1.0%)		
Sputum, n (%)						
Never/only with chest infections	237 (74.3%)	276 (86.5%)	277 (87.1%)	277 (92.0%)	0.0079	<0.0001
Few days per months	76 (23.8%)	40 (12.5%)	36 (11.3%)	24 (8.0%)		
≥Several days per week	6 (1.9%)	3 (0.9%)	5 (1.6%)	0 (0%)		
Wheeze, n (%)					0.0124	0.0124
Never/only with chest infections	295 (92.5%)	303 (95.0%)	306 (95.9%)	295 (98.0%)		
Few days per months	21 (6.6%)	16 (5.02%)	12 (3.8%)	5 (1.7%)		
≥Several days per week	3 (0.9%)	0 (0%)	1 (0.31%)	1 (0.3%)		
Any respiratory symptom≥monthly, n (%)	185 (60.0%)	98 (30.72%)	94 (29.5%)	59 (19.6%)	0.0003	<0.0001
Impact of chest on activities, n (%)					0.0003	<0.0001
Does not stop activities	156 (48.9%)	257 (80.6%)	288 (90.3%)	275 (91.4%)		
Prevents 1–2 activities	133 (41.7%)	50 (15.7%)	29 (9.1%)	22 (7.3%)		
Prevents most/all activities	30 (9.4%)	12 (3.8%)	2 (0.6%)	4 (1.3%)		
Impact of chest on work, n (%)					0.0006	<0.0001
Does not affect work	194 (60.8%)	282 (88.4%)	291 (92.7%)	288 (95.7%)		
Interferes with/made me change work	112 (35.1%)	34 (10.7%)	22 (7.01%)	13 (4.3%)		
Made me stop work	13 (4.1%)	3 (0.9%)	1 (0.32%)	0 (0%)		
Breathless at rest/during personal care, n (%)	1 (0.3%)	0 (0%)	0 (0%)	0 (0%)	1	0.3173
Walks slower than peers/stops for rest at own pace, n (%)	83 (26.2%)	53 (16.6%)	25 (7.8%)	16 (5.3%)	<0.0001	<0.0001
Breathless on hills, n (%)	136 (42.9%)	70 (21.9%)	43 (13.5%)	38 (12.6%)	0.0014	<0.0001
*Quality of life, median score (IQR)*						
SGRQ total score	9 (1–23)	0 (0–7)	1.1 (0–2.86)	0 (0–1.44)	0.0018	<0.0001
SGRQ symptoms score	10 (3–22)	3 (0–14)	4.4 (0–9.6)	0 (0–6.3)	<0.0001	<0.0001
SGRQ activity score	11 (0–35)	0 (0–6)	0 (0–0)	0 (0–0)	0.0016	<0.0001
SGRQ impact score	6 (0–15)	0 (0–4)	0 (0–0)	0 (0–0)	<0.0001	<0.0001
**Care seeking**		**12 months after TB-treatment completion**		**Last visit**		
Any self-reported acute respiratory OPD visit in the last 12 months‡, n (%)	n/a	49 (15.4%)	n/a	27 (8.9%)	n/a	0.0271
Number of respiratory OPD visits in those that sought care‡, n (%)					**n/a**	0.0007
1		40 (80.6%)		17 (63.0%)		
2		6 (12.2%		9 (33.3%)		
>3	n/a	3 (6.1%)	n/a	1 (3.7%)		
Admission for ill health in last 12 months‡, n (%)	n/a	21 (5.8%)	n/a	13 (4.4%)	n/a	0.1779
Admissions for respiratory reason in last 12 months‡, n (%)	n/a	9 (2.8%)	n/a	3 (0.9%)	n/a	0.0833
Retreated for TB in last 12 months‡, n (%)	n/a	8 (2.51%)	1 (0.3%)	2 (0.7%)	n/a	0.0578

n/a: indicated data for comparison were not collected at this time point.

*Pairwise comparisons between TB-treatment completion and 12-month data using McNemar’s test for categorical variables and Wilcoxon rank sum for continuous variables.

†$Symptom questions derived from SGRQ.

‡Data reported from the end of the first 12 months and end of 3 years of the study.

BMI, body mass index; OPD, outpatient department.

Among those completing the extended follow-up, unscheduled healthcare seeking for a respiratory complaint (one or more episodes) was reported by 49/319 (15.3%) in their first year after completing TB treatment, falling to 27 (8.9%) participants in the last year of the study, p=0.03, of which the majority (17/27) reported only one episode. Within this group, 11/319 participants were retreated for TB: 8 were in the first year after treatment completion, 1 between the first and second year and 2 between the second and third years after treatment completion.

### Spirometry

The proportion of participants contributing ATS standard spirometry was 285/319 (89.3%) at TB-treatment completion, 292/319 (91.5%) at 1 year, 293/319 (91.8%) at 2 years and 272/301 (90.3%) at the final 3-year visit.

On average, spirometry parameters improved over time. The mean FVC z-score significantly improved from −0.91 (1.21) to −0.64 (1.10) over the 3-year period, p<0.001 (table 3). The FEV_1_ z-score improved from −1.07 (1.24) to 0.91 (1.16), p<0.001. Posthoc analysis examined the hypothesis that most change took place in the first year. Online supplemental tables 8E and 9E show that compared with a smooth linear change, a two-segment fitted model had had significantly improved predictive capability by Bayesian Information Criteria (p<0.001), demonstrating significantly higher recovery of lung function between treatment completion and 9 months, and less change later.

**Table 3:** Change in spirometry (completed to ATS standard) over the 3-year post-TB-treatment completion

Variable	TB-treatment completion N=285	1-year post-TB treatment completion N=292	2-year post-TB treatment completion N=293	3-year post TB-treatment completion N=272	Mean change over 3 years (normalised to time)	P value comparing TB-treatment completion and 3-year visit
Spirometry*						
FVC z-Score, mean (SD)	−0.91 (1.21)	−0.61 (1.11)	−0.55 (1.10)	−0.64 (1.10)	0.28 (0.63)	<0.0001
FEV_1_ z-score, mean (SD)	−1.07 (1.24)	−0.87 (1.18)	−0.81 (1.15)	−0.91 (1.16)	0.16 (0.59)	<0.0001
FEV_1_/FVC ratio z-score, mean (SD)	−0.38 (1.26)	−0.52 (1.30)	−0.56 (1.34)	−0.56 (1.30)	−0.19 (0.69)	<0.0001
Reversible†	4 (1.25%)	7 (2.40%)	4 (1.37%)	1 (0.37%)		0.1800

*ATS standard spirometry n=285/319 at TB-treatment completion=292/319 at 1-year visit n=293/319 at 2-year visit, n=272/301 at the 3-year last visit.

†Reversible spirometry with an FEV_1_ change of 12%.

FEV1, forced expiratory volume in 1 second; FVC, forced vital capacity.

**Figure 2:**
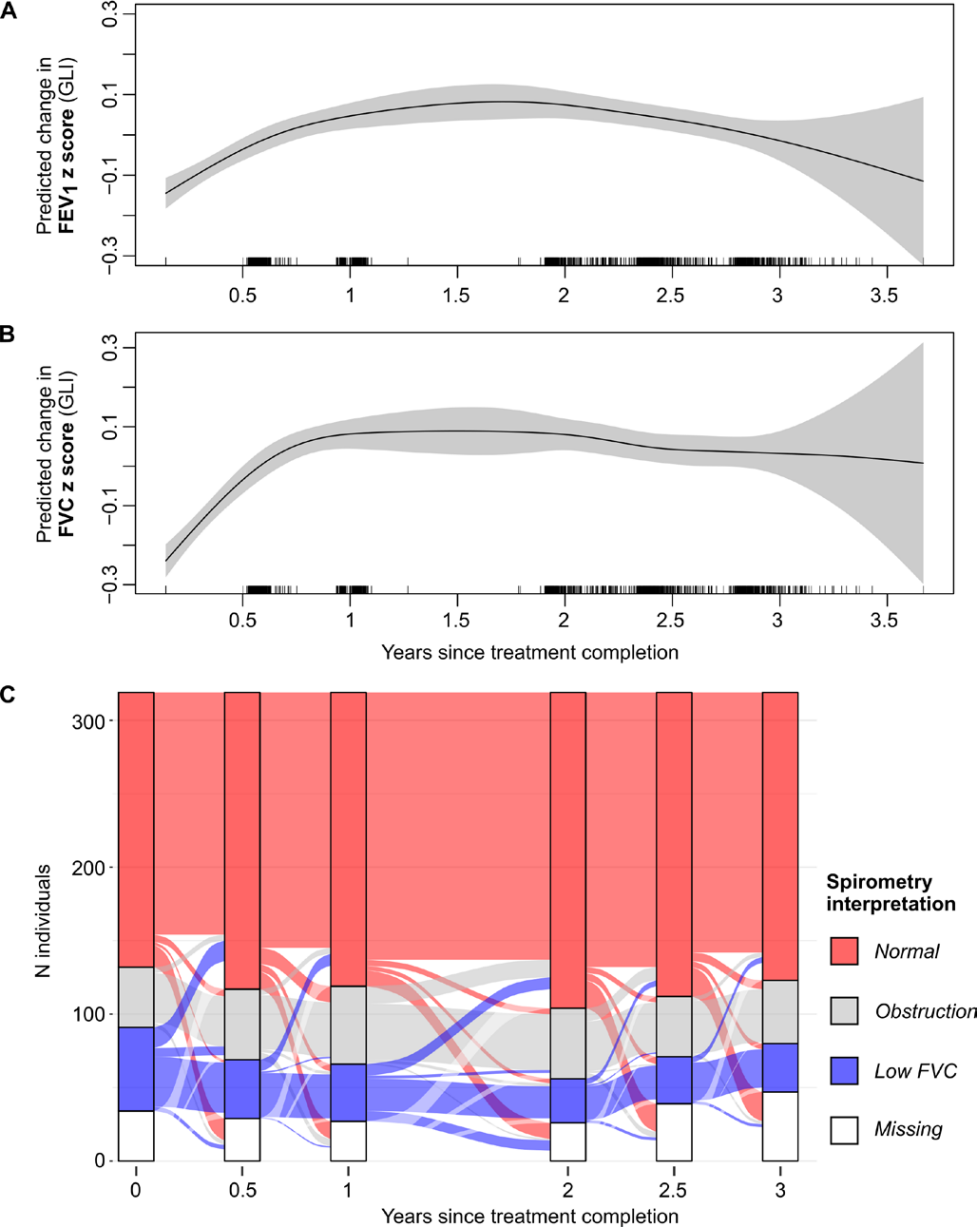
(A) FEV1 z-score change (non-normalised individual intervals). (B) FVC z-score change (non-normalised individual intervals). (C) Sankey plot showing change of spirometry pattern (using GLI and LLN in those who completed ATS standard spirometry) between each visit from TB treatment completion to the final 3-year visit. FEV1, forced expiratory volume in 1 second; FVC, forced vital capacity.

Categorical interpretation of spirometry using both LLN and fixed ratio (GOLD) cut-offs was concordant with these findings: the proportion of participants with ‘low FVC’ using these two definitions decreased from 57/285 (20.0%) to 33/271 (12.1%) and 70/285 (24.7%) to 40 (14.7%), respectively (p<0.001 for both, online supplemental table 3E). Overall, these categorical changes occurred mostly in the first 6 months, although transitioning from low FVC to normal or obstructive patterns is seen over the whole period (figure 2).

### Change in clinical measures over time stratified by lung function at TB-treatment completion

Despite improvements in symptoms and lung function at the group level, some individuals demonstrated worsening of FEV_1_ and FVC of over 100 mL from treatment completion to the 3-year visit (73/246, 29.7% and 52/246, 21.1%, respectively, table 4). The distribution in lung function change is asymmetric, and non-normal on visual inspection as shown in online supplemental figure 2E, providing some evidence for the existence of a proportion of patients with deteriorating spirometry. We therefore examined how participant outcomes at 3 years might be predicted by earlier spirometry.

**Table 4:** Proportion of participants experiencing clinically relevant improvement, deterioration or change in health markers

Parameter*	Classification of change	Improvement	No change	Deterioration
BMI	Change≥1.46 kg/m^2^	106 (36.2%)	168 (57.3%)	19 (6.5.%)
		2.79 (2.15 to 4.34)	0.20 (−0.30 to 0.80)	−2.04 (−2.77 to −1.52)
SGRQ	Change≥4 units	158 (53.2%)	127 (42.8%)	12 (4.04%)
		−18.17 (−26.53 to 9.91)	0 (−1.84 to 0)	17.01 (10.80 to 19.81)
6 min walk test distance	Change≥26 m	142 (53.6%)	72 (27.2%)	51 (19.3%)
		75.28 (49.95 to 102.09)	3.48 (−8.23 to 12.85)	(−85.96 to −41.23)
6 min walk test desaturation (SpO_2_ less 92% at end of walk)	Change ‘yes’ or ‘no’	14 (5.26%)	248 (93.2%)	4 (1.50%)
Presence of monthly symptoms	Change between present/absent monthly symptoms	125 (41.5%)	166 (55.2%)	10 (3.3%)
FEV1 (L)†	Change≥100 mL	88 (35.8%)	85 (34.6%)	73 (29.7%)
		0.21 (0.15 to 0.31)	0 (−0.04 to 0.06)	−0.18 (−0.31 to − 0.14)
FVC (L)†	Change≥100 mL	126 (51.2%)	68 (27.6%)	52 (21.1%)
		0.25 (0.17 to 0.42)	0 (−0.03 to 0.05)	−0.25 (−0.34 to −0.15)
Unscheduled visit to healthcare for respiratory condition	Change≥1 visit	46 (15.4%)	246 (82.3%)	7 (2.3%)
		−1 (−1 to −1)	0 (0 to 0)	2 (2 to 2)

Measure between baseline visit and last visit at 3 years (n=309).

*All continuous variable normalised to time (1095 days since baseline measurements).

†Matched postbronchodilator spirometry to ATS standards, n=246.

BMI, body mass index; FEV1, forced expiratory volume in 1 second; FVC, forced vital capacity.

When stratified by spirometry pattern at treatment completion, by 3-year follow-up, 68% (34/50) of those with ‘low FVC’ had improved by over 0.1 L (median change 0.31 L, SD 0.24–0.50). Only 4/50 (8.0%) experienced further deterioration (online supplemental table 2E).

In those with a pattern of obstructive spirometry at TB treatment completion, 40.6% (15/37) had an improvement in FEV_1_ of over 0.1 L (median 0.17 L, SD 0.13–0.29) at 3 years. Deterioration of over 0.1 L in FEV_1_ was noted in 10/37 (27.0%), with median change of −0.24 L (−0.39 to −0.15). FVC also deteriorated in 29.7% (11/37) of this group, with a median deterioration of 0.16 L (−0.18 to −0.16) over the 3-year period.

Among those with normal spirometry at TB-treatment completion 47.8% (76/159) had a greater than 0.1 L improvement in FVC (median change 0.23 L, IQR 0.17–0.36) and 28.9% (46/159) of participants experienced an improvement in their FEV_1_ (median change 0.21 L, IQR 0.15–0.27). However, 37/159 (23.3%) had decline in FVC of greater than 0.1 L over the 3-year follow-up (median change −0.25 L (−0.34 to −0.16)) and 34.6% (55/159) had a decline in FEV_1_ greater than 0.1 L with a median decline of 0.18 L (−0.31 to −0.12) (online supplemental table 2E).

### Predictors of lung function over 3 years

**Figure 3:**
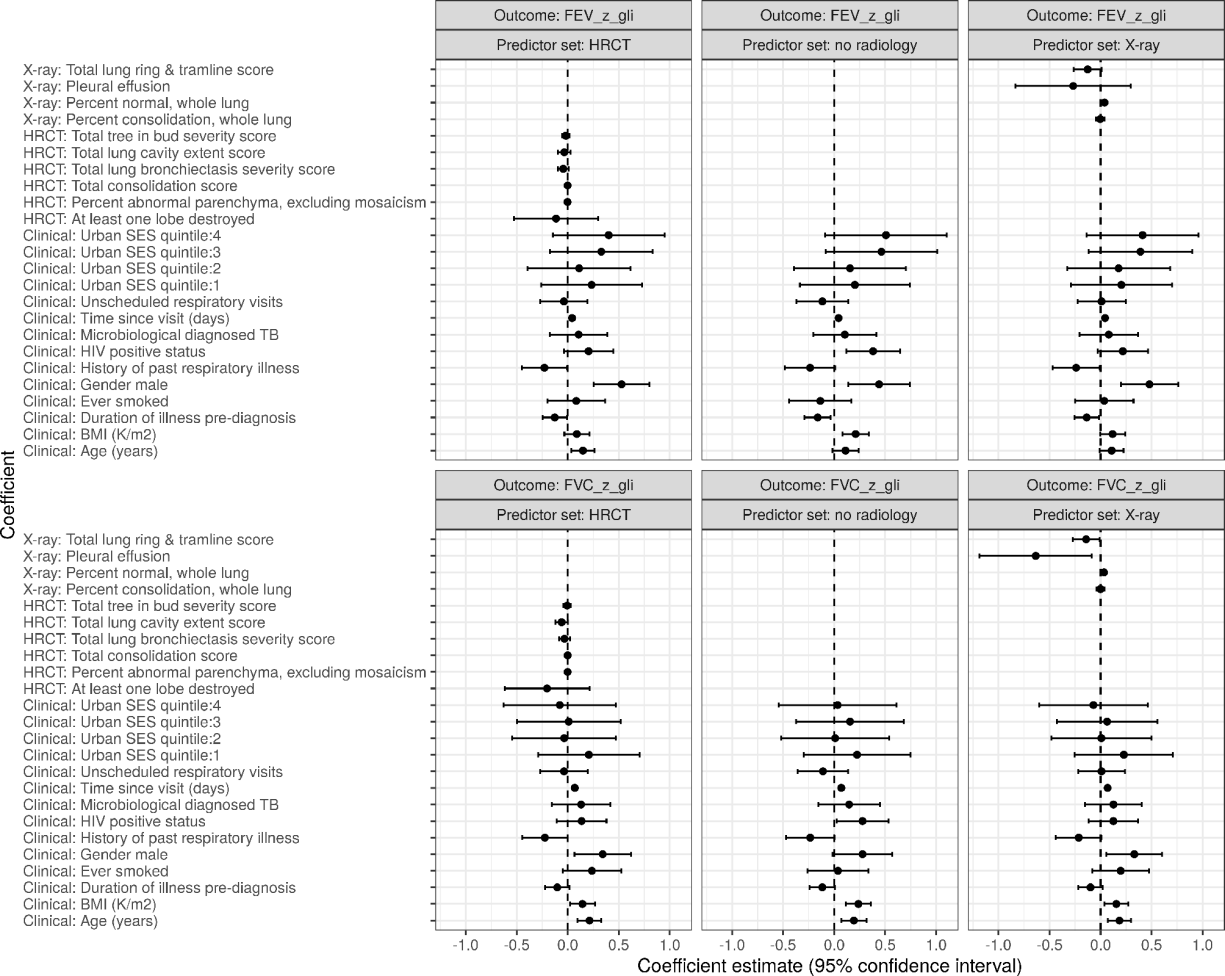
Coefficient estimates (95% CI) for multivariable regression models with Z-score change in spirometry values in the 3-year follow-up period after TB treatment completion as the outcome and different covariate sets. FEV, forced expiratory volume; FVC, forced vital capacity; SES, socioeconomic status.

Using linear mixed effects models adjusted for age, sex and time, and without any radiological variables, being HIV-positive was associated with greater mean FEV_1_ z score (0.38) at 3 years (95% 0.12 to 0.64), as was having a higher body mass index (BMI) at TB treatment completion (0.21 L/kg/m^2^, 95% CI 0.08 to 0.34) (figure 3 and online supplemental table 4E). When including radiological variables, the only significant predictor of FEV_1_ at 3 years other than age and sex was the duration of illness prior to TB diagnosis. Longer duration was associated with a lower mean FEV_1_ z-score (−0.16, 95% 0.29 to −0.03) at 3 years, with this association found in all z-score models but not in absolute values (figure 3 and online supplemental tables 6E, 7E and 5E, respectively).

In adjusted mixed effects models considering FVC at 3 years as the outcome, positive associations were noted in those with HIV coinfection (z-score, 0.28, 95% CI 0.02 to 0.53) and higher BMI at TB-treatment completion (0.24, 95% CI 0.11 to 0.36). FVC-z scores were higher in older participants and males. BMI was associated with a higher FVC z-score in the model without radiology only (0.24, 95% CI 0.11 to 0.36), the percentage of normal lung on X-ray was associated with a higher FVC z-score (0.03, 95% CI 0.01 to 0.05) whereas a higher ring and tramline score indicating radiological bronchiectasis was associated with lower FVC-z score (−0.14, 95% CI −0.27 to −0.01) at 3 years.

Factors associated with absolute change in FEV_1_ z-scores between TB-treatment completion and the 3-year visit were age (−0.011, 95% CI 0.02 to −0.01) and being HIV positive at TB treatment completion (0.21, 95% 0.005 to 0.37). In models which included HRCT, FEV_1_ z-score change was predicted by history of past respiratory illness (0.15, 95% CI 0.01 to 0.30). Percentage consolidation on X-ray (0.03, 95% 0.01 to 0.06) at TB treatment completion and pleural effusion on X-ray (0.42, 95% CI 0.07 to 0.77) also predicted higher FEV_1_ change over the 3 years.

Increasing age (0.01, 95% CI −0.02 to 0.00) and HIV positive status (0.18, 95% CI 0.01 to 0.35) were also significant predictors of change in FVC over the 3 years (online supplemental table 6E). In models which included HRCT, FVC z-score change was predicted by the total ‘tree in bud’ score (0.03, 95% CI 0.00 to 0.50) and the extent of consolidation (0.01, 95% CI 0.00 to 0.01). Similarly to FEV_1_ z-scores, percentage consolidation on X-ray (0.05, 95% 0.02 to 0.07) at TB treatment completion and pleural effusion on X-ray (0.39, 95% CI 0.04 to 0.74) also predicted higher FVC change over 3 years. The same patterns of predictors were seen in models describing the change in absolute FEV and FVC over time (online supplemental table 5E).

## Discussion

In this cohort of adults successfully completing PTB treatment, we described an initial high burden of lung disease in which 60% still reported one or more respiratory symptoms and 34.4% had abnormal spirometry at the point of TB treatment completion.^4^ In this paper, we show that 3 years after treatment completion, most people had significantly improved symptoms (19.6% experiencing symptoms), with most improvement in the first year. However, 27.9% had abnormal spirometry, mostly obstruction (15.8%). When looking at patterns of change over time, the majority of the cohort experienced no change or an improvement in FEV_1_ and FVC; however, a proportion had substantive (over 0.1 L) decline in spirometry (FEV_1_ 29.7% and FVC 21.1%), which was not limited to those that had abnormal spirometry at treatment completion. Lung function at 3 years was higher in those who had higher BMI and those who were HIV positive at treatment completion. The extent of tree-in-bud pathology on HRCT and parenchymal change or pleural effusion on CXR predicted improvement in lung function over 3 years, while more extensive ring and tramline change at treatment completion was associated with lower FVC z-score at 3 years.

The high burden of PTB lung disease reported here is consistent with other literature and replicated the findings from our original cohort.^5 12–15^ Symptoms were more common than reports from the wider population in Blantyre (19.6% v 11.8%), as was obstructive lung function abnormality (15.8% v 4.2%).^16^ While the effect of PTB on lung function is well documented, reports on how this changes over time are sparse. In spontaneously healed patients with pulmonary TB in South Korea, FEV_1_ decline overtime has been demonstrated to exceed that of uninfected individuals.^17^ Among South African miners, an increasing number of prior PTB episodes was associated with a greater deficit in both FEV_1_ and FVC compared with miners without a TB history.^17 18^ A separate study of HIV-positive patients with TB in South Africa, estimated a 35 mL excess FEV_1_ loss per year and a 57 mL excess FVC loss.^19^

In adulthood, an average decline of 30 mL a year in FEV_1_ is normal.^20^ In this cohort recovering from PTB, the majority experienced an improvement in FEV_1_ of 30 mL in 3 years (or 10 mL/year). It is worth noting this was following an acute respiratory insult, where recovery in the short term would be expected after treatment. However. 29.7% of all participants experienced a decline over 100 mL over the 3 years (or 33 mL/year), and among this group, the median decline in FEV_1_ was 180 mL (or 60 mL/year), which is similar to those with tobacco-related chronic obstructive pulmonary disease (COPD).^21 22^ The primary driver of this decline is unclear from the models we developed. We did not include smoking in our model, as cumulative smoking levels were low, limiting our capacity to estimate tobacco risks. However, possible other drivers may include chronic colonisation with other pathogens, nutritional status or environmental factors such as exposure to biomass.

We saw the burden of low FVC decrease over 3-year follow-up, and the burden of obstruction non-statically significantly increased. In our participants, this change in pattern appears to represent significant improvement in FVC compared with FEV_1_, which may reflect recovery rather than disease progression. Other studies have reported a change to an obstructive pattern from a restrictive pattern, with suggestions that this may be due to progression of small airways pathology during the healing process.^23^

Our finding that HIV positive participants had higher FEV_1_ and FVC at 3 years is consistent with our observations at TB treatment completion.^5^ Although the majority of participants were on antiretroviral therapy (ART), many were severely immunosuppressed even by the point of PTB treatment completion, which may suggest that impaired immune response to mycobacteria during the acute PTB phase is associated with less lung destruction.^24^ However, as an overall group, we have previously shown that there was still a high burden of PTLD seen among HIV-infected participants, which may be associated with immune reconstitution within the HIV coinfected participants included in this study, many of whom initiated ART close to treatment for PTB.^5 25^

Our findings support the finding that a proportion of TB survivors have persistent symptoms and poor lung function at 3 years after treatment. Negative HIV status, low BMI and extensive lung parenchymal change appears to predict lung function decline, but abnormal spirometry at the end of TB-treatment does not. Interestingly, CXR appears to be a better predictor of mean 3-year lung function than HRCT. In LMIC, it may be that a simple score of ‘normal’ lung on CXR is both helpful and more accessible than HRCT.

To the best of our knowledge, we are the first group to have recorded symptoms, spirometry and radiological findings prospectively over a 3-year period post PTB-treatment completion. While our study is limited by the lack of a control group, previous cross-sectional research has shown the effect of TB on lung function, and our focus here was on within-individual changes over time. Limitations also include recruitment from a single site which may limit generalisability, and recall bias related to prior illness and recent healthcare usage. There may have been selection bias at initial enrolment, or during follow-up, with those with poor or declining health less likely to attend study visits and therefore relatively under-represented in our study. However, strengths of this study include prospective recruitment, and high rates of retention (76% of participants for 3 years) for a study of this duration and type.

In summary, we found that the majority of patients with postpulmonary-TB experienced improved symptoms and lung function. A smaller proportion continued to have a substantial burden of symptoms and lung function abnormalities 3 years after TB treatment completion. This population is likely to become larger as TB survival improves.^26^ While there is understandably a focus on treatment of active TB disease, patients who have suboptimal recovery have limited access to care and few treatment options.^3^ Formal guidance on individual patient management of health and well-being post-TB for those who have suboptimal recovery are lacking but would be welcomed by clinicians.^27^ Guideline development should be underpinned by research to determine what interventions would be clinically effective and cost-effective.^28^ The potential for improving the health, psychosocial and economic status among this group of overlooked patients is substantial. However, interventions at the health system level are unlikely to be prioritised without a deeper understanding of the drivers of residual symptoms and lung function decline, and their economic ill-effects. For those who fail to quickly recover following TB infection, treatment options might include pulmonary rehabilitation, as for other chronic respiratory diseases. Identifying those with the poorest functional or physiological improvement at an earlier stage would ensure that this most affected group are not left behind.

## Data Availability

Data are available on reasonable request. Data are available on reasonable request to the corresponding author.
