# Supplementary material for: Respiratory symptoms and lung function in patients treated for pulmonary tuberculosis in Malawi: a prospective cohort study
Source: Thorax. 2021 Dec 22;77(11):1131–9. doi: 10.1136/thoraxjnl-2021-217190 (PMC9606518; doi:10.1136/thoraxjnl-2021-217190)
Supplement: Supplementary data [file thoraxjnl-2021-217190supp001.pdf]

Supplement Tables: Change in pulmonary function and respiratory outcomes in patient treated for pulmonary tuberculosis in Malawi: A longitudinal cohort study

Table 1E: GOLD definitions

Findings	Spirometric Definition
Post-bronchodilator mild obstruction	FEV_1/FVC ratio <0.7
Post-bronchodilator moderate obstruction	FEV_1/FVC ratio <0.7 and FEV_1 <80% and FEV_1 >50% predicted*
Post-bronchodilator severe obstruction	FEV_1/FVC ratio <0.7 and FEV_1 <50% predicted*
Spirometric restriction	FEV_1/FVC ratio >0.7 and FVC<80% predicted*
Airway reversibility	FEV_1 increase >200ml and >12% following bronchodilator
Predicted values based on age, sex and height referenced to GLI as reported in this study.	

Figure 1E: Direct acyclic graph (DAG) of variable included in regression models.

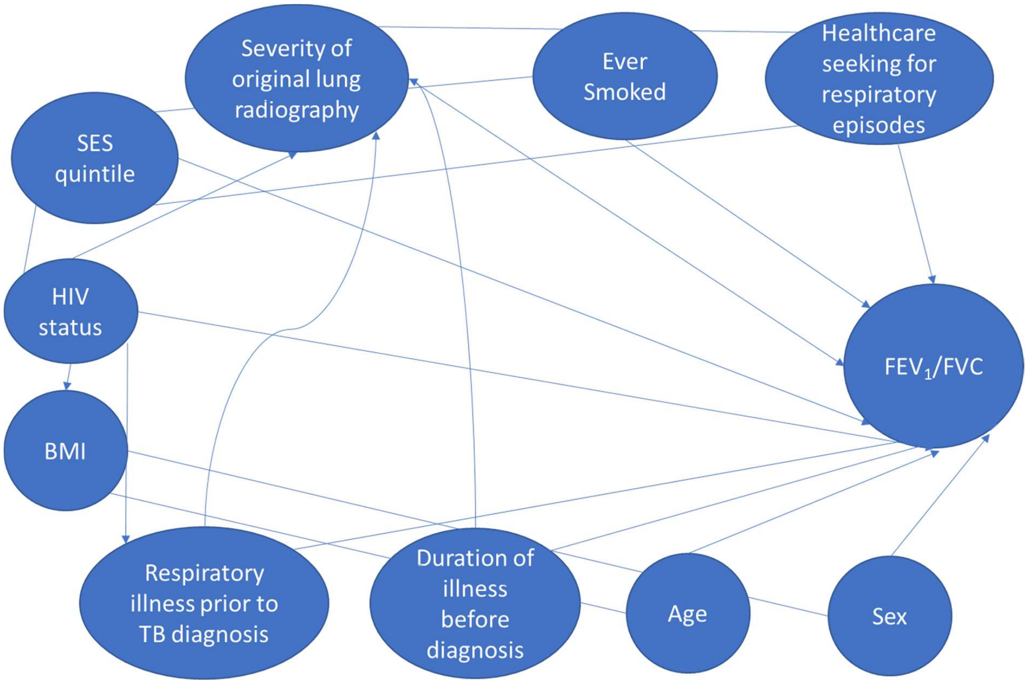

Radiological variables were: % abnormal parenchyma, excluding mosaicism (HRCT),At least 1 lobe destroyed (HRCT).Total lung bronchiectasis dilatation severity score (HRCT),Total tree in bud severity score (HRCT),Total lung cavities extent (number score 0-18) (HRCT),Total consolidation score, across whole lung (HRCT), % normal, across whole lung (X-ray), % consolidation, across whole lung (X-ray), total lung ring & tramline score (X-ray), pleural effusion (X-ray).

Figure 2E: FEV/FVC change over time – density plots of the absolute difference in z-score between completion of treatment and three-year measurement.

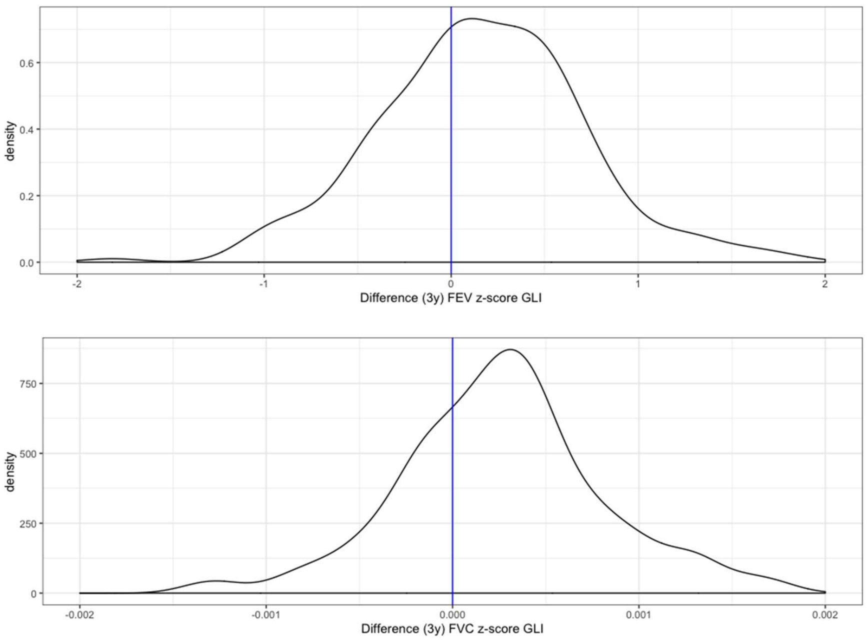

Table 2E: Proportion of participants experiencing clinically relevant improvement, deterioration or change in health markers between TB-treatment completion visit and last visit at 3 years.

Stratified to those who had Low FVC (LLN) at TB-treatment completion n = 55				
**Parameter**	**Classification of change**	**Improvement**	**No change**	**Deterioration**
BMI	Change ≥1.46kg/m^{2+}	15 (27.8%)<br>2.47 (1.9-4.9)	34 (63.0%)<br>0.24 (-0.3-0.8)	5 (9.3%)<br>-1.70 (-2.0- -1.7)
SGRQ	Change ≥4 units $^{++}$	36 (65.5%)<br>-20.90 (-27.1- -15.8)	17 (30.9%)<br>0 (-0.98- 0)	2 (3.6%)<br>20.12 (18.6-21.7)
6 minute walk test distance	Change ≥26m $^{++}$	24 (55.8%)<br>80.18 (46.3-94.5)	11 (25.6%)<br>6.021 (-18.2-12.4)	8 (18.60%)<br>-54.18 (-93.0—40.9)
6 minute walk test desaturation (spo2 less 92% at end of walk)	Change "yes" or "no"	2 (4.4%)	43 (95.6%)	0
Presence of monthly symptoms	Change between present / absent monthly symptoms	29 (52.73%)	24 (43.6%)	2 (3.64%)
FEV1 (litres)$	Change ≥100ml $^{++}$	27 (54.0%)<br>0.24 (0.17-0.39)	15 (30.0%)<br>0 (-0.06- 0.02)	8 (16.0%)<br>-0.16(-0.18- -0.16)
FVC (litres)$	Change ≥100ml $^{++}$	34 (68.0%)<br>0.31 (0.24-0.50)	12 (24.0%)<br>0.03 (-0.01-0.07)	4 (8.0%)<br>0.32(-0.34- --0.26)
Unscheduled visit to healthcare for respiratory condition	Change ≥1 visit	12 (22.2%)<br>-1 (-1 - -1)	41 (75.9%)<br>0 (0-0)	1 (1.85%)<br>2 (2-2)
**Stratified to those who had obstruction at TB-treatment completion n =39**				
BMI	Change ≥1.46kg/m^2	11 (28.2%)<br>2.78 (1.91-3.13)	24 (61.6%)<br>0.30 (-0.05-1.00)	4 (10.3%)<br>-1.55 (-2.23—1.50)
SGRQ	Change ≥4 units	25 (65.8%)<br>-19.28 (-35.6- -14.1)	12 (31.6%)<br>-0.95 (-2.0-0.4)	1 (2.6%)<br>12.73 (12.7-12.7)
6 minute walk test distance	Change ≥26m	12 (33.3%)<br>84.75 (55.74-122.15)	15 (41.7%)<br>-3.10 (-14.27-15.25)	9 (25.05%)<br>-67.14 (-114.46- -51.47)
6 minute walk test desaturation (spo2 less 92% at end of walk)	Change "yes" or "no"	5 (14.71%)	29 (85.3%)	0
Presence of monthly symptoms	Change between present / absent monthly symptoms	21 (53.9%)	18 (46.2%)	0
FEV1 (litres)*	Change ≥100ml	15 (40.6%)<br>0.17 (0.13-0.29)	12 (32.4%)<br>0.04 (-0.01-0.80)	10 (27.0%)<br>-0.24 (- -0.39- -0.15)
FVC (litres)	Change ≥100ml	16 (43.2%)<br>0.20 (0.16-0.45)	10 (27.0%)<br>-0.02 (-0.05-0.0)	11 (29.7%)<br>-0.16 (-0.31- -0.12)
Unscheduled visit to healthcare for respiratory condition	Change ≥1 visit	7 (18.4%)	30 (79.0%)	1 (2.6%)
**Stratified by those who had normal TB-treatment completion spirometry n=176**				
BMI	Change ≥1.46kg/m^2	68 (40.0%)<br>3.02 (2.86-4.59)	95 (55.9%)<br>0.09 (-0.50-0.68)	7 (4.1%)<br>-2.63 (-3.1—1.51)
SGRQ	Change ≥4 units	86 (49.1%)<br>-15.73 (-26.5- -8.36)	80 (45.7%)<br>0 (-1.8-0.0)	9 (5.1%)<br>16.09 (10.2-19.61)
6 minute walk test distance	Change ≥26m	93 (58.1%)<br>75.90 (50.83-103.96)	39 (24.45)<br>3.91 (-6.06-12.02)	28 (17.5%)<br>-53.88 (-82.51- -40.99)
6 minute walk test desaturation (spo2 less 92% at end of walk)	Change "yes" or "no"	5 (3.1%)	152 (94.4%)	4 (2.5%)

Presence of monthly symptoms	Change between present / absent monthly symptoms	66 (37.5%)	104 (59.1%)	6 (3.4%)
FEV1 (litres)$	Change ≥100ml	46 (28.9%)<br>0.21 (0.15-0.27)	58 (36.5%)<br>0 (-0.05-0.06)	55 (34.6%)<br>-0.18 (-0.31- -0.12)
FVC (litres)	Change ≥100ml	76 (47.8%)<br>0.23 (0.17-0.36)	46 (28.9%)<br>0.02 (-0.02-0.05)	37 (23.3%)<br>-0.25 (-0.34- -0.16)
Unscheduled visit to healthcare for respiratory condition	Change ≥1 visit	24 (13.7%)	146 (83.4%)<br>0 (0-0)	5 (2.9%)<br>2 (2-2)

$ Matched post bronchodilator spirometry to ATS standards n=50 *Matched post bronchodilator spirometry to ATS standards n=37 &Matched post bronchodilator spirometry to ATS standards n=159

+ No existing MCID agreed in literature: cut-off calculated by 0.5 x standard deviation of baseline data and as previously reported (4)

++ Derived from COPD literature, normal lung function change in Malawi adults and previously reported baseline data(4, 17, 26).

Table 3E: Change in spirometry (completed to ATS standard) over the 3-years post TB-treatment completion including lower limit of normal (LLN), GOLD and z-scores

Variable	TB-treatment completion N=285	1-years post TB treatment completion N=292	2-years post TB treatment completion N=293	3-years post TB-treatment completion N=272	Mean change over 3-years (normalised to time)	P value comparing TB-treatment completion and 3-year visit
**Spirometry***						
FVC, mean L (SD)	3.17 (0.77)	3.30 (0.77)	3.33 (0.77)	3.24 (0.79)	0.10 (0.29)	<0.0001
FEV_1, mean L (SD)	2.56 (0.68)	2.63 (0.67)	2.64 (0.66)	2.57 (0.66)	0.03 (0.24)	0.1159
FVC Z-Score, mean (SD)	-0.91 (1.21)	-0.61 (1.11)	-0.55 (1.10)	-0.64 (1.10)	0.28 (0.63)	<0.0001
FEV_1 Z-score, mean (SD)	-1.07 (1.24)	-0.87 (1.18)	-0.81 (1.15)	-0.91 (1.16)	0.16 (0.59)	<0.0001
FEV_1/FVC ratio z-score, mean (SD)	-0.38 (1.26)	-0.52(1.30)	-0.56 (1.34)	-0.56 (1.30)	-0.19 (0.69)	<0.0010
**Spirometry pattern (GOLD)**						
Normal, n (%)	187 (65.6%)	211 (72.3%)	218 (74.4%)	196 (72.1%)		0.0412
Mild obstruction, n (%)	9 (3.16%)	5 (1.7%)	11 (3.8%)	8 (2.9%)		
Moderate obstruction, n (%)	14 (4.91%)	25 (8.6%)	25 (8.5%)	25 (9.2%)		
Severe/very severe obstruction, n (%)	5 (1.75%)	6 (2.1%)	4 (1.37%)	3 (1.1%)		
Low FVC, n (%)	70 (24.6%)	45 (15.4%)	35 (12.0%)	40 (14.7%)		<0.0001
**Spirometry pattern (LLN)**						
Normal, n (%)	187 (65.6%)	200 (68.5%)	215 (73.4%)	196 (72.1%)		0.3938
Obstruction, n (%)	41 (14.4%)	53 (18.2%)	46 (16.4%)	43 (15.8%)		
Low FVC, n (%)	57 (20.0%)	39 (13.4%)	30 (10.3%)	33 (12.1%)		<0.0004
Reversable†	4 (1.25%)	7 (2.40%)	4 (1.37%)	1 (0.37%)		P=0.1800

*ATS standard spirometry n=285/319 at TB-treatment completion=292/319 at 1-year visit n=293/319 at 2-year visit, n=272/301 at the 3-year last visit.

† Reversable spirometry with an FEV_1 change of 12%.

Table 4E: Association between covariates and spirometry outcomes by linear mixed effects regression in investigating spirometry values in the three year follow up period after TB treatment completion using *absolute values* among individuals who completed 3-year follow up.

Variable measured at TB treatment completion	Multivariable model no radiology	Multivariable model (HRCT)	Multivariable model (X-ray)
Outcome: Mean absolute FEV1 (L) over the 3-year follow up period			
Time since visits (days)	0.004 (-0.004-0.011)	0.004 (-0.004-0.011)	0.004 (-0.003-0.011)
Age (years)	-0.164 (-0.226- - 0.103)*	-0.145 (-0.203- -0.087)*	-0.165 (-0.223- -0.107)*
Gender male	0.859 (0.71-1.00)*	0.897 (0.760-1.034)*	0.880 (0.740-1.019)*
Microbiological diagnosed TB	0.044 (-0.106-0.194)	0.052 (-0.09-0.194)	0.034 (-0.108-0.175)
HIV positive status	0.185 (0.058-0.312)*	0.113 (-0.008-0.233)	0.119 (-0.003-0.242)
History of past respiratory illness	-0.103 (-0.221-0.016)	-0.100(-0.21- -0.011)	-0.104 (-0.219-0.009)
Urban SES quintile<br>-2nd poorest<br>-Middle<br>-2nd most wealthy<br>-Most wealthy	 0.062 (-0.197-0.323)<br>0.038 (-0.227-0.303)<br>0.223 (-0.041-0.487)<br>0.294 (0.006-0.582)	 0.084 (-0.163-0.333)<br>0.026 (-0.227-0.279)<br>0.179 (-0.074-0.431)<br>0.252 (-0.022-0.527)	 0.060 (0.479- -0.185)<br>0.048 (-0.202-0.298)<br>0.193 (-0.057-0.440<br>0.252 (-0.004-0.117)
Ever smoked	-0.031 (-0.179-0.117)	0.049 (-0.093-0.191)	0.038 (-0.103-0.181)
BMI (Kg/m2)	0.093 (0.031-0.155) *	0.042 (-0.019-0.103)	0.056 (-0.004-0.117)
Unscheduled respiratory visits	-0.088 (-0.211-0.035)	-0.060 (-0.176-0.054)	-0.037 (-0.154-0.079)
Duration of illness pre-diagnosis	-0.058 (-0.120-0.003)	-0.038 (-0.098-0.021)	-0.044 (-0.104-0.015)
% abnormal parenchyma, excluding mosaicism (HRCT)		-0.001 (-0.002- -0.001)*	
At least 1 lobe destroyed (HRCT)		-0.046 (-0.253-0.160)	
Total lung bronchiectasis dilatation severity score (HRCT)		-0.023 9-0.050-0.003)	
Total tree in bud severity score (HRCT)		-0.003 (-0.021-0.014)	
Total lung cavities extent/number score (HRCT)		-0.003 (-0.033-0.028)	
Total consolidation score, across whole lung (HRCT)		-0.001 (-0.005-0.003)	
% normal, across whole lung (X-ray)			0.014 (0.004-0.024)*
% consolidation, across whole lung (X-ray)			-0.009 (-0.03-0.011)
Total lung ring & tramline score (X-ray)			-0.046 (-0.11-0.021)
Pleural effusion (X-ray)			-0.086 (-0.366-0.195)
Outcome: Mean absolute FVC (L) over 3-year follow up period			
Time since visits (days)	0.024 (0.015-0.033)*	0.024 (0.015-0.033)*	0.024 (0.015-0.032)*
Age (years)	-0.087 (-0.157- -0.016)*	-0.073 (-0.142- -0.005)*	-0.090 (-0.158- -0.023)*
Gender male	1.033 (0.867-1.200)*	1.067 (0.904-1.231)*	1.064 (0.903-1.226)*
Microbiological diagnosed TB	0.074 (-0.097 -0.246)	0.077 (-0.091-0.245)	0.064 (-0.100-0.229)
HIV positive status	0.177 (0.031-0.323)*	0.113 (-0.031-0.257)	0.109 (-0.033-0.251)
History of past respiratory illness	-0.129 (-0.266- 0.007)	-0.124 (-0.256-0.008)	-0.120 (-0.253-0.012)
Urban SES quintile<br>-2nd poorest<br>-Middle<br>-2nd most wealthy<br>-Most wealthy	 0.075 (-0.224-0.374)<br>-0.027 (-0.330-0.277)<br>0.122 (-0.208-0.452)<br>0.122 (-0.207-0.452)	 0.083 (-0.212-0.377)<br>-0.039 (-0.340-0.262)<br>0.063 (-0.238-0.363)<br>0.079 (-0.248-0.406)	 0.074 (-0.211-0.359)<br>0.028 (-0.318-0.262)<br>0.070 (-0.221-0.361)<br>0.070 (-0.25-0.386)
Ever smoked	0.081 (-0.089-0.250)	0.160 (-0.010-0.329)	0.152 (-0.0121-0.317)
BMI (K/m2)	0.127 (0.056-0.199)*	0.0842 (0.011-0.157)*	0.090 (0.020-0.161)*
Unscheduled respiratory visits	-0.105 (-0.246-0.004)	-0.079 (-0.217-0.059)	-0.051 (-0.187-0.084)
Duration of illness pre-diagnosis	-0.041 (-0.113-0.029)	-0.027 (-0.098-0.005)	-0.029 (-0.098-0.040)
% abnormal parenchyma, excluding mosaicism (HRCT)		-0.001 (-0.002- -0.000)*	
At least 1 lobe destroyed (HRCT)		-0.101 (-0.347-0.146)	
Total lung bronchiectasis dilatation severity score (HRCT)		-0.024 (-0.056-0.007)	

Total tree in bud severity score (HRCT)		0.002 (-0.019-0.023)	
Total lung cavity extent score (0-18) (HRCT)		-0.012 (-0.049-0.002)	
Total consolidation score, across whole lung (HRCT)		-0.013 (-0.049-0.024)	
% normal, across whole lung (X-ray)			0.012 (0.001-0.024)*
% consolidation, across whole lung (X-ray)			-0.001 (-0.034-0.013)
Total lung ring & tramline score (X-ray)			-0.059 (-0.138-0.019)
Pleural effusion (X-ray)			-0.286 (-0.611-0.040)

* Statistically significant at p<0.05 level. All variables included in a model are reported

Table 5E: Estimates of association between covariates and spirometry outcomes by linear mixed effects regression, investigating *absolute change* in spirometry values in the three-year follow up period after TB treatment completion among individuals who completed 3-year follow up.

Variable measured at TB treatment completion	Multivariable model no radiology	Multivariable model (HRCT)	Multivariable model (X-ray)
Outcome: Difference in absolute FEV1 (L) from baseline to 3-year follow up period (negative is worsening FEV_1)			
Age (years)	-0.006 (-0.009- -0.003)*	-0.006 (-0.009- -0.003)*	-0.005 (-0.089- -0.003)*
Gender male	0.063 (-0.013-0.140)	0.06 (-0.157-0.138)	0.051 (-0.23-0.125)
Microbiological diagnosed TB	-0.002 (-0.080-0.76)	-0.009 (-0.874-0.069)	0.004 (-0.071-0.079)
HIV positive status	0.083 (0.017-0.150)*	0.093 (0.027-0.160)*	0.103 (0.037-0.128)*
History of past respiratory illness	0.056 (-0.004-0.118)	0.061 (0.001-0.123)	0.040 (-0.020-0.100)
Urban SES quintile<br>-2nd poorest<br>-Middle<br>-2nd most wealthy<br>-Most wealthy	 -0.029 (-0.172-0.113)<br>0.006 (-0.140-0.150)<br>-0.039 (-0.186-0.107)<br>-0.071 (-0.231-0.087)	 -0.012 (-0.154-0.129)<br>0.22 (-0.123-0.168)<br>-0.008 (-0.156-0.140)<br>-0.049 (-0.209-0.110)	 -0.006 (-0.144-0.131)<br>0.028 (-0.112-0.168)<br>0.001 (-0.141-0.142)<br>-0.026 (-0.180-0.128)
Ever smoked	0.034 (-0.043-0.11)	0.008 (-0.070-0.087)	0.04 (-0.04-0.115)
BMI (K/m2)	-0.003 (-0.133-0.008)	-0.001 (-0.011-0.011)	-0.003 (-0.013-0.008)
Unscheduled respiratory visits	-0.007 (-0.061-0.465)	-0.027 (-0.082-0.286)	-0.028 (-0.081-0.026)
Duration of illness pre-diagnosis	0.000 (-0.000- 0.000)	0.000 (-0.000-0.000)	0.000 (-0.000- 0.000)
% abnormal parenchyma, excluding mosaicism (HRCT)		0.000 (-0.000-0.000)	
At least 1 lobe destroyed (HRCT)		-0.047 (-0.162-0.684)	
Total lung bronchiectasis dilatation severity score (HRCT)		0.112 (-0.017-0.039)	
Total tree in bud severity score (HRCT)		0.006 (-0.004-0.016)	
Total lung cavities extent/number score (HRCT)		0.003 (-0.015-0.202)	
Total consolidation score, across whole lung (HRCT)		0.002 (-0.001-0.004)	
% normal, across whole lung (X-ray)			-0.001 (-0.007-0.004)
% consolidation, across whole lung (X-ray)			0.010 (-0.001-0.021)
Total lung ring & tramline score (X-ray)			-0.003 (-0.019-0.013)
Pleural effusion (X-ray)			0.160 (0.016-0.304)
Outcome: Difference in absolute FVC (L) from baseline to 3-year follow up period (negative is worsening FVC)			
Age (years)	-0.007 (-0.010- -0.004)*	-0.007 (-0.011- -0.004)*	-0.007 (-0.010- -0.033)*
Gender male	0.107 (0.213-0.192)*	0.112 (0.030-0.194)*	0.089 (0.009-0.170)*
Microbiological diagnosed TB	0.037 (-0.51-0.124)	0.036 (-0.048-0.120)	0.046 (-0.037-0.127)
HIV positive status	0.104 (0.030-0.180)*	0.128 (0.056-0.200)*	0.133 (0.063-0.202)*
History of past respiratory illness	0.049 (-0.020-0.119)	0.553 (-0.010-0.121)	0.035 (-0.031-0.101)
Urban SES quintile<br>-2nd poorest<br>-Middle<br>-2nd most wealthy<br>-Most wealthy	 0.016 (-0.144-0.177)<br>0.030 (-0.134-0.193)<br>-0.018 (-0.183-0.146)<br>-0.041 (-0.220-0.137)	 0.042 (-0.109-0.195)<br>0.045 (-0.111-0.202)<br>0.022 (-0.137-0.181)<br>-0.011 (-0.183-0.160)	 0.056 (-0.093-0.205)<br>0.057 (-0.09-0.208)<br>0.036 (-0.118-0.189)<br>0.029 (-0.137-0.195)
Ever smoked	0.103 (0.017-0.190)*	0.055 (-0.028-0.139)	0.104 (0.022-0.186)*
BMI (K/m2)	-0.006 (-0.018-0.006)	0.001 (-0.011-0.127)	-0.004 (-0.160-0.007)
Unscheduled respiratory visits	0.014 (-0.046-0.076)	-0.019 (-0.079-0.040)	-0.022 (-0.080-0.035)
Duration of illness pre-diagnosis	0.000 (-0.000-0.000)	0.000 (-0.000-0.001)	-0.000 (-0.000-0.000)
% abnormal parenchyma, excluding mosaicism (HRCT)		0.000 (-0.000-0.001)	
At least 1 lobe destroyed (HRCT)		0.047 (-0.077-0.171)	
Total lung bronchiectasis dilatation severity score (HRCT)		0.007 (-0.23-0.037)	
Total tree in bud severity score (HRCT)		0.013 (0.002-0.024)*	
Total lung cavities extent/number score (HRCT)		0.005 (-0.014-0.024)	
Total consolidation score, across whole lung (HRCT)		0.004 (0.001-0.006)*	

% normal, across whole lung (X-ray)			-0.004 (-0.010-0.002)
% consolidation, across whole lung (X-ray)			0.016 (0.004-0.028)*
Total lung ring & tramline score (X-ray)			-0.007 (-0.024-0.010)
Pleural effusion (X-ray)			0.180 (0.024-0.337)*

* All variables included in a model are reported

Table 6E: Linear regression model, investigating *z-score change* in spirometry values in the three-year follow up period after TB treatment completion

Variable measured at TB treatment completion	Multivariable model no radiology	Multivariable model (HRCT)	Multivariable model (X-ray)
Difference in Z-sore FEV1 (L) from baseline to 3-year follow up period (negative is worsening FEV_1)			
Age (years)	-0.011 (-0.02- -0.01)*	-0.01 (-0.02- -0.01)*	-0.01 (-0.02- -0.00)
Gender male	0.09 (-0.09-0.28)	0.08 (-0.11-0.26)	0.06 (-0.13-0.24)
Microbiological diagnosed TB	-0.03 (-0.23-0.16)	-0.062 (-0.25-0.13)	-0.02 (-0.21-0.16)
HIV positive status	0.19 (0.02-0.16)*	0.21 (0.05-0.37)*	0.24 (0.07-0.39)*
History of past respiratory illness	0.14 (-0.01-0.29)	0.15 (0.01-0.30)*	0.09 (-0.06-0.24)
Urban SES quintile<br>-2nd poorest<br>-Middle<br>-2nd most wealthy<br>-Most wealthy	 -0.07 (-0.42-0.27)<br>0.00 (-0.35-0.35)<br>-0.07 (-0.43-0.29)<br>-0.23 (-0.62-0.17)	 -0.3 (-0.37-0.32)<br>0.7 (-0.28-0.42)<br>0.02 (-0.34-0.38)<br>-0.16 (-0.54-0.22)	 -0.01 (-0.35-0.32)<br>0.07 (-0.28-0.41)<br>0.03 (-0.32-0.37)<br>-0.12 (-0.49-0.26)
Ever smoked	0.10 (-0.09-0.29)	0.04 (-0.151-0.23)	0.12 (-0.8-0.29)
BMI (K/m2)	-0.00 (-0.03-0.02)	0.00 (-0.02-0.03)	-0.00 (-0.3-0.3)
Unscheduled respiratory visits	-0.04 (-0.18-0.09)	-0.09 (-0.22-0.04)	-0.08 (-0.22-0.05)
Duration of illness pre-diagnosis	0.00 (-0.00-0.00)	0.00 (-0.00-0.00)	0.00 (-0.00-0.00)
% abnormal parenchyma, excluding mosaicism (HRCT)		0.00 (0.00-0.00)	
At least 1 lobe destroyed (HRCT)		-0.19 (-0.47-0.08)	
Total lung bronchiectasis dilatation severity score (HRCT)		0.02 (-0.5-0.09)	
Total tree in bud severity score (HRCT)		0.02 (-0.01-0.04)	
Total lung cavities extent/number score (HRCT)		-0.00 (-0.04-0.04)	
Total consolidation score, across whole lung (HRCT)		0.01 (-0.01-0.01)	
% normal, across whole lung (X-ray)			0.00 (-0.012-0.14)
% consolidation, across whole lung (X-ray)			0.03 (0.01-0.06)*
Total lung ring & tramline score (X-ray)			0.00 (-0.04-0.4)
Pleural effusion (X-ray)			0.42 (0.07-0.77)*
Difference in Z-score FVC (L) from baseline to 3-year follow up period (negative is worsening FVC)			
Age (years)	-0.01 (-0.02—0.00)*	-0.01 (-0.02- -0.01)*	-0.01 (-0.02- -0.00)*
Gender male	0.13 (-0.07-0.33)	0.13 (-0.05-0.31)	0.08 (-0.10-0.26)
Microbiological diagnosed TB	0.07 (-0.13-0.28)	0.07 (-0.12-0.25)	0.09 (-0.09-0.27)
HIV positive status	0.18 (0.01-0.35)*	0.23 (0.71-0.40)*	0.24 (0.09-0.40)*
History of past respiratory illness	0.15 (-0.01-0.31)	0.17 (0.02-0.31)	0.13 (-0.21-0.27)
Urban SES quintile<br>-2nd poorest<br>-Middle<br>-2nd most wealthy<br>-Most wealthy	 -0.00 (-0.37-0.37)<br>-0.01 (-0.38-0.36)<br>-0.04 (-0.42-0.33)<br>-0.21 (-0.62-0.20)	 0.06 (-0.28-0.40)<br>0.04 (-0.31-0.39)<br>0.61 (-0.30-0.42)<br>-0.13 (-0.51-0.26)	 0.10 (-0.23-0.44)<br>0.06 (-0.28-0.40)<br>0.09 (-0.25-0.44)<br>-0.03 (-0.41-0.34)
Ever smoked	0.23 (0.03-0.43)*	0.12 (-0.07-0.31)	0.22 (0.04-0.41)
BMI (K/m2)	-0.01 (-0.4-0.01)	0.00 (-0.02-0.03)	-0.01 (-0.04-0.02)
Unscheduled respiratory visits	0.01 (-0.13-0.14)	-0.07 (-0.21-0.06)	-0.08 (-0.21-0.05)
Duration of illness pre-diagnosis	-0.00 (-0.00-0.00)	0.00 (-0.00-0.00)	-0.00 (-0.00-0.00)
% abnormal parenchyma, excluding mosaicism (HRCT)		0.00 (-0.00-0.00)	
At least 1 lobe destroyed (HRCT)		0.103 (-0.18-0.38)	
Total lung bronchiectasis dilatation severity score (HRCT)		0.00 (-0.07-0.07)	
Total tree in bud severity score (HRCT)		0.03 (0.00-0.50)*	
Total lung cavities extent/number score (HRCT)		0.01 (-0.4-0.5)	
Total consolidation score, across whole lung (HRCT)		0.01 (0.00-0.01)*	
% normal, across whole lung (X-ray)			-0.01 (-0.02-0.01)
% consolidation, across whole lung (X-ray)			0.05 (0.02-0.07)*
Total lung ring & tramline score (X-ray)			-0.02 (-0.06-0.02)
Pleural effusion (X-ray)			0.39 (0.04-0.74)*

* All variables included in a model are reported

Table 7E: Association between covariates and spirometry outcomes by linear mixed effects regression in the 3-year follow up period after TB treatment completion using z-scores among individuals who completed 3-year follow up.

Variable measured at TB treatment completion	Multivariable model no radiology	Multivariable model (HRCT)	Multivariable model (X-ray)
Outcome: Z-score FEV_1 over three follow up period			
Time since visits (days)	0.04 (0.03-0.06)*	0.04 (0.03-0.06)*	0.04 (0.03-0.06)*
Age (years)	0.11 (-0.02-0.23)	0.15 (0.03-0.26)*	0.11 (-0.01-0.23)
Gender male	0.44 (0.14-0.74)*	0.53 (0.25-0.80)*	0.48 (0.20-0.76)*
Microbiological diagnosed TB	0.10 (-0.21-0.41)	0.11 (-0.17-0.29)	0.080 (-0.21-0.37)
HIV positive status	0.38 (0.12-0.64)*	0.20 (-0.04-0.44)	0.22 (-0.03-0.47)
History of past respiratory illness	-0.24 (-0.48-0.01)	-0.23 (-0.45- -0.01)*	-0.24 (-2.03- -0.47)
Urban SES quintile			
-2nd poorest	0.20 (-0.33-0.74)	0.23 (-0.26-0.73)	0.20 (-2.03- -0.47)
-Middle	0.16 (-0.39-0.70)	0.11 (-0.39-0.61)	0.18 (-0.33-0.68)
-2nd most wealthy	0.46 (-0.08-1.01)	0.33 (-0.17-0.83)	0.39 (-0.11-0.89)
-Most wealthy	0.51 (-0.08-1.10)	0.40 (-0.14-0.94)	0.41 (-0.14-0.96)
Ever smoked	-0.14 (-0.44-0.17)	0.08(-0.20-0.36)	0.03 (-0.25-0.32)
BMI (K/m2)	0.21 (0.08-0.34)*	0.09 (-0.03-2.11)	0.12 (-0.01-0.24)
Unscheduled respiratory visits	-0.12 (-0.37-0.13)	-0.04 (-0.25-0.19)	0.01(-0.22-0.24)
Duration of illness pre-diagnosis	-0.16 (-0.29- -0.03)*	-0.13 (-0.24- -0.01)*	-0.13 (-0.23- -0.01)*
% abnormal parenchyma, excluding mosaicism (HRCT)		-0.00 (-0.00- -0.00)*	
At least 1 lobe destroyed (HRCT)		-0.11 (-0.52-0.29)	
Total lung bronchiectasis dilatation severity score (HRCT)		-0.04 (-0.09-0.01)	
Total tree in bud severity score (HRCT)		-0.02 (-1.03-0.05)	
Total lung cavities extent/number score (HRCT)		-0.03 (-0.09-0.03)	
Total consolidation score, across whole lung (HRCT)		-0.00 (-0.43- -0.01)	
% normal, across whole lung (X-ray)			0.04 (0.01-0.06)*
% consolidation, across whole lung (X-ray)			-0.00 (-0.05-0.04)
Total lung ring & tramline score (X-ray)			-0.13 (-0.26- 0.01)
Pleural effusion (X-ray)			-0.26 (-0.83-0.29)
Outcome: Z-score FVC over three follow up period			
Time since visits (days)	0.07 (0.05-0.09)*	0.07 (0.05-0.09)*	0.07 (0.04-0.08)*
Age (years)	0.19 (0.07-0.32)*	0.21(0.09-0.32)*	0.19 (0.07-0.30)*
Gender male	0.28 (-0.01-0.57)	0.34 (0.06-0.61)*	0.33 (0.05-0.60)*
Microbiological diagnosed TB	0.15 (-0.15-0.45)	0.13 (-0.5-0.41)	0.13 (-0.15-0.40))
HIV positive status	0.28 (0.02-0.53)*	0.14 (-0.10-0.37)	0.13(-0.11-0.36)
History of past respiratory illness	-0.23 (0.47-0.00)	-0.22 (-0.44-0.01)	-0.21 (-0.43-0.01)
Urban SES quintile			
-2nd poorest	0.22 (-0.30-0.75)	0.21 (-0.29-0.70)	0.23 (-0.25-0.71)
-Middle	0.01 (-0.52-0.54)	-0.04 (-0.54-0.47)	0.01 (-0.48-0.50)
-2nd most wealthy	0.16 (-0.37-0.68)	0.01 (-0.50-0.51)	0.06 (-0.43-0.56)
-Most wealthy	0.03 (-0.54-0.61)	-0.07 (-0.62-0.47)	-0.07 (-0.60-0.46)
Ever smoked	0.04 (-0.26-0.34)	0.24 (-0.05-0.52)	0.20 (-0.08-0.48)
BMI (K/m2)	0.24 (0.11-0.36)*	0.14 (0.02-0.26)	0.15 (0.04-0.27)
Unscheduled respiratory visits	-0.11 (0.36-0.14)	-0.04 (-0.27-0.19)	0.01 (-0.22-0.24)
Duration of illness pre-diagnosis	-0.12 (-0.24-0.01)	-0.10 (-0.22-0.02)	-0.10 (-0.22-0.01)
% abnormal parenchyma, excluding mosaicism (HRCT)		-0.00 (-0.00- -0.00)*	
At least 1 lobe destroyed (HRCT)		-0.20 (-0.61-0.21)	
Total lung bronchiectasis dilatation severity score (HRCT)		-0.03 (-0.08-0.02)	
Total tree in bud severity score (HRCT)		-0.00 (0.04-0.02)	
Total lung cavities extent/number score (HRCT)		-0.06 (-0.12-0.00)	
Total consolidation score, across whole lung (HRCT)		-1.92 (-0.01-0.01)	
% normal, across whole lung (X-ray)			0.03 (0.01-0.05)*
% consolidation, across whole lung (X-ray)			-0.00 (-0.04-0.03)
Total lung ring & tramline score (X-ray)			-0.14 (-0.27- -0.01)*
Pleural effusion (X-ray)			-2.27 (-1.18- --0.09)

Table 8E: Statistical evaluation of the rates of change in FVC within the follow-up period.

From visual inspection of the graph showing FEV_1 and FVC z-scores over time (Figure 2, main manuscript), there appeared to be a rapid improvement in the first year after TB treatment completion, and diminished rate of improvement after this. To test this hypothesis, we *post hoc examined the a*ssociations between pre-specified predictors and FEV_1 and FVC over time. W used a linear mixed-effects models, fitting a random effect for participant and adjusting for time from TB-treatment completion as a fixed effect. Time from TB treatment completion was also modelled as piecewise linear and using a natural spline in adjusted mixed effects models. The latter "piecemeal" model introduces an inflexion point at 9 months (inferred from visual inspection of the graph), with an independent fixed effect in the periods before and after this point.

Compared the piecewise and linear models, the former had better Akaike's Information Criteria (2726.523 compared with 2762.627), better Bayesian Information Criteria (2851.21 compared with 2865.629), and improved log likelihood (-1340.26 vs -1362.31, p<0.0001).

**FVC**									
**Linear model**					**Piecewise model**				
term	est	95% CI lower	95% CI upper	p	term	est	95% CI lower	95% CI lower	p
					Years since TB treatment completion < 0.75 y	0.25	0.14	0.36	<0.0001
Years since TB treatment completion	0.063	0.04	0.09	<0.0001	Year since TB treatment completion > 0.75 y	0.05	0.03	0.08	<0.0001
Age (z score)	0.17	0.05	0.30	0.0054	Age (z score)	0.17	0.05	0.29	0.0058
Male sex	0.29	0.00	0.58	0.0504	Male sex	0.29	0.00	0.58	0.0496
Microbiology confirmed TB	0.15	-0.15	0.45	0.3212	Microbiology confirmed TB	0.15	-0.15	0.45	0.3251
HIV-positive	0.32	0.06	0.57	0.0148	HIV-positive	0.32	0.06	0.57	0.0150
History of past respiratory illness	-0.21	-0.45	0.03	0.0805	History of past respiratory illness	-0.21	-0.45	0.03	0.0838
Urban SES quintile - 2nd poorest	0.26	-0.26	0.78	0.3224	Urban SES quintile - 2nd poorest	0.27	-0.25	0.78	0.3148
Urban SES quintile - Middle	0.06	-0.47	0.58	0.8300	Urban SES quintile - Middle	0.06	-0.47	0.59	0.8229
Urban SES quintile - 2nd most wealthy	0.19	-0.33	0.71	0.4761	Urban SES quintile - 2nd most wealthy	0.19	-0.33	0.72	0.4731
Urban SES quintile - Most wealthy	0.04	-0.53	0.62	0.8828	Urban SES quintile - Most wealthy	0.05	-0.53	0.62	0.8741
Ever smoked tobacco	0.10	-0.19	0.40	0.4988	Ever smoked tobacco	0.10	-0.20	0.40	0.5053
BMI (z score)	0.22	0.10	0.35	0.0004	BMI (z score)	0.22	0.10	0.35	0.0004
Unscheduled respiratory visits	-0.10	-0.34	0.15	0.4369	Unscheduled respiratory visits	-0.09	-0.34	0.15	0.4528
Duration of illness pre diagnosis (z score)	-0.12	-0.24	0.002	0.0545	Duration of illness pre diagnosis (z score)	-0.12	-0.25	0.002	0.0541

Table 9E: Statistical evaluation of the rates of change in FEV_1 within the follow-up period. As for 6A, modelling FEV_1 as the dependent variable.

Compared the piecewise and linear models, the former had better AIC (2580.875 compared with 2600.424), and improved log likelihood (-1267.44 vs -1281.21, p<0.0001), although worse BIC when penalised for added parameters (2705.562 compared with 2703.426).

FEV									
**Linear model**					**Piecewise model**				
term	est	95% CI lower	95% CI upper	p	term	est	95% CI lower	95% CI lower	p
					Years since TB treatment completion < 0.75 y	0.15	0.05	0.26	0.0041
Years_visit	0.04	0.02	0.06	0.0005	Year since TB treatment completion > 0.75 y	0.04	0.01	0.06	0.0021
Age (z score)	0.09	-0.04	0.22	0.1586	Age (z score)	0.09	-0.04	0.22	0.1563
Male sex	0.45	0.15	0.75	0.0031	Male sex	0.46	0.15	0.76	0.0031
Microbiology confirmed TB	0.10	-0.21	0.41	0.5328	Microbiology confirmed TB	0.10	-0.21	0.41	0.5227
HIV-positive	0.42	0.15	0.68	0.0020	HIV-positive	0.41	0.15	0.68	0.0021
History of past respiratory illness	-0.22	-0.47	0.02	0.0760	History of past respiratory illness	-0.22	-0.47	0.02	0.0745
Urban SES quintile - 2nd poorest	0.20	-0.34	0.74	0.4618	Urban SES quintile - 2nd poorest	0.20	-0.34	0.73	0.4760
Urban SES quintile - Middle	0.16	-0.38	0.71	0.5530	Urban SES quintile - Middle	0.16	-0.39	0.70	0.5743
Urban SES quintile - 2nd most wealthy	0.46	-0.08	1.01	0.0952	Urban SES quintile - 2nd most wealthy	0.46	-0.09	1.00	0.1007
Urban SES quintile - Most wealthy	0.48	-0.12	1.07	0.1147	Urban SES quintile - Most wealthy	0.47	-0.12	1.07	0.1195
Ever smoked tobacco	-0.11	-0.41	0.20	0.4867	Ever smoked tobacco	-0.11	-0.42	0.19	0.4693
BMI (z score)	0.21	0.08	0.33	0.0017	BMI (z score)	0.21	0.08	0.34	0.0015
Unscheduled respiratory visits	-0.11	-0.37	0.14	0.3749	Unscheduled respiratory visits	-0.11	-0.37	0.14	0.3819
Duration of illness pre diagnosis (z score)	-0.16	-0.29	-0.03	0.0140	Duration of illness pre diagnosis (z score)	-0.16	-0.29	-0.03	0.0139

Table 10E: Comparison of limited demographics stratified by HIV at baseline, 1 year and 3 years showing very similar characteristics of the cohort at each time point.

	**Baseline**			**1 year**			**3 years**		
	**Total**	**HIV-**	**HIV+**	**Total**	**HIV-**	**HIV+**	**Total**	**HIV-**	**HIV+**
	n=405**	n=159 (39.3%)	n=244 (60.2%)	n=319*	n=126 (39.4%)	n=192 (60.2%)	n=301*	n=118 (39.2%)	n=182 (60.5%)
Age at baseline, median years) (IQR)†	35 (28–41)	30 (24-37)	37 (32-42)	35 (29-41)	31 (24-38)	37 (32-42)	34 (28-41)	31 (24-38)	37 (32-42)
Male sex	275 (67.9%)	115 (72.3%)	158 (64.8%)	212 (66.2%)	91 (72.2%)	120 (62.5%)	200 (66.4%)	86 (72.9%)	113 (62.1%)

* n=1 unknown

** n= 2 unknown

† Age is given corrected for the age at baseline, irrespective of the when the follow-up was performed to allow comparison.
